# Correspondence: Guillain-Barré syndrome and hemorrhagic fever with renal syndrome

**DOI:** 10.1186/s12879-019-4216-8

**Published:** 2019-08-05

**Authors:** Linpei Jia, Rufu Jia, Hongliang Zhang

**Affiliations:** 1 Department of Nephrology, Xuanwu Hospital of Capital Medical University, Changchun Street 45#, Beijing, 100053 China; 2 Central Hospital of Cangzhou, Xinhua Middle Street 201#, Cangzhou, 061001 Hebei Province China; 3 Department of Life Sciences, the National Natural Science Foundation of China, Shuangqing Road 83#, Beijing, 100085 China

**Keywords:** Hemorrhagic fever with renal syndrome, Guillain-Barré syndrome, Critical illness polyneuropathy, Albumino-cytologic dissociation

## Abstract

Jiao and colleagues reported a case of hemorrhagic fever with renal syndrome who developed respiratory failure and symmetrical flaccid paralysis of all extremities. Electrophysiology revealed peripheral nerve injuries mainly in axons. They reached a diagnosis of Guillain-Barré syndrome (GBS) associated with hemorrhagic fever with renal syndrome. Although the case is interesting, the diagnosis of GBS in such a patient should be with caution. Critical illness polyneuropathy (CIP) is an important and common differential diagnosis of GBS, especially in intensive care settings. Differentiating CIP from the axonal variants of GBS may be difficult on purely clinical grounds. Albumino-cytologic dissociation in CSF can help differentiate GBS from other disorders.

## Background

Jiao and colleagues reported a case of hemorrhagic fever with renal syndrome who developed respiratory failure and symmetrical flaccid paralysis of all extremities [1]. Electrophysiology revealed peripheral nerve injuries mainly in axons. They reached a diagnosis of Guillain-Barré syndrome (GBS) associated with hemorrhagic fever with renal syndrome. Although the case is interesting, the diagnosis of GBS in such a patient should be with caution.

GBS is an immune-mediated disorder of the peripheral nervous system and respiratory failure requiring mechanical ventilation is a serious complication of GBS [2]. However, the reported patient developed respiratory failure and refractory shock first, and then received mechanical ventilation. As per the description provided by the authors, the patient presented with symmetrical flaccid paralysis of all extremities but normal spontaneous respiration after cease of sedation, analgesia and muscle relaxants, on day 12 [1]. Usually, respiratory muscles are easily involved in a quadriplegic GBS patient. Of note is that the patient was absent from diplopia or facioplegia. In this regard, critical illness polyneuropathy (CIP) should be highly suspected.

CIP and critical illness myopathy (CIM) are frequent complications of critical illness involving both of the motor and sensory axons [3, 4]. The cardinal clinical sign of CIP and CIM presents as flaccid and symmetric weakness of extremities as well as respiratory muscles [3, 5]. Patients with CIP may show a distal sensory loss, e.g. pain, temperature, or vibration [3, 5]. CIP and/or CIM occur in approximately 70% of patients with sepsis or systemic inflammatory response syndrome (SIRS) [2, 5], 60% of acute respiratory distress syndrome [2, 5], and up to 100% of patients with multiple organ failure [2, 5]. CIP and CIM alone, or in combination prolong weaning from mechanical ventilation and physical rehabilitation [3, 5].

We previously reported a case of critical illness polyneuropathy and myopathy with reversible tetraplegia after percutaneous nephrostolithotomy and septic shock [6]. The axonal variants of GBS might be difficult to distinguish from CIP due to the similar clinical manifestations and electrophysiological signs. Although the clinical presentation and electrophysiology would not give supportive discrimination, cerebral spinal fluid (CSF) might be of help. A clinical hallmark of GBS is albumino-cytologic dissociation in CSF [7]. In this case, the patient failed to receive a lumbar puncture. Therefore, the differential diagnosis of CIP cannot be ruled out.

In summary, CIP is an important and common differential diagnosis of GBS, especially in intensive care settings. Differentiating CIP from the axonal variants of GBS may be difficult on purely clinical grounds. Albumino-cytologic dissociation in CSF can help differentiate between these two diseases.

## Data Availability

Not applicable.

## Abbreviations

CIM: Critical illness myopathy
CIP: Critical illness polyneuropathy
CSF: Cerebral spinal fluid
GBS: Guillain-Barré syndrome
SIRS: Systemic inflammatory response syndrome
