# Supplementary material for: Correspondence: Guillain-Barré syndrome and hemorrhagic fever with renal syndrome
Source: BMC Infect Dis. 2019 Aug 5;19:687. doi: 10.1186/s12879-019-4216-8 (PMC6683354; doi:10.1186/s12879-019-4216-8)
Supplement: Related texts

# Reply: Authors’ response

**Authors:** Lei Wu, Xuan Sun, Jie Hu

Zhang and colleagues suggested a reasonable doubt about the patient’s diagnosis that we should take critical illness polyneuropathy (CIP) or critical illness myopathy (CIM), instead of Guillain–Barré syndrome (GBS), into consideration based on the scenario of critical illness. Although it is difficult to distinguish CIP/CIM and GBS because of their similar clinical manifestations (such as axonal motor and sensory polyneuropathy), some factors should be taken into account when we deal with this dilemma.

First, prolongation of F-waves on electromyography (EMG) is the most important feature of GBS other than CIP/CIM [8]. Nerve root impairment is one of the characteristics of GBS, and it usually appears as prolongation of F-waves on EMG [9] and albuminocytological dissociation in cerebrospinal fluid (CSF). Of note is absence of prolongation of F-waves is one of the diagnostic criteria for CIP [8]. In our case, the patient showed absence of F wave in right tibial nerve and prolongation of F-wave in left tibial nerve [1], which is a strong supportive evidence of GBS.

Second, the lack of evidence of albuminocytological dissociation should not be an exclusion criterion for GBS. Based on our current knowledge, only 64% of patients with GBS have the feature with albuminocytological dissociation [10]. According to the recently proposed new diagnostic classification system, GBS diagnosis can be decided with clinical ground and EMG manifestations, without the need for additional laboratory tests, and whether or not they fulfill existing diagnostic criteria [11].

Third, we cannot arbitrarily exclude the diagnosis of GBS due to the absence of cranial nerve deficits, such as diplopia or facioplegia. GBS is highly diverse in terms of the presence, distribution and extent of cranial nerve deficits, sensory symptoms, weakness, ataxia, and the course of the disease [10]. Only 50% of the GBS patients present cranial nerve deficits [12].

Fourth, the presence or absence of respiratory problems cannot be used in differential diagnosis of ICUAW and GBS since respiratory muscle weakness also occurs commonly in patients with CIP/CIM [13].

Fifth, good response to intravenous immunoglobin (IVIG) therapy strongly supported the diagnosis of GBS. For GBS, IVIG or plasma exchange (PE) is the optimal management approach, alongside supportive care [14]. CIP and CIM are pathophysiologic definition describing the weak critically ill patient with evidence of associated neuromuscular dysfunction, which represent the most prominent causes of intensive care unit acquired weakness (ICUAW). In intensive settings, sepsis, shock, hyperglycemia, the presence and degree of multiorgan failure, immobilization, and the utilization of steroids or neuromuscular blocker are the potential risk factors of ICUAW [15]. Unlike GBS, there are no proven therapies that could reverse ICUAW, i.e., CIP and CIM [8]. Treatment of underlying disease such as sepsis and shock, control of hyperglycemia, minimization of sedation, and early mobilization might be potential therapeutic strategies for ICUAW [15].

Last, etiological agents might highlight the diagnosis of GBS. Wachira et al. found that virus (such as cytomegalovirus and influenza virus) and Campylobacter jejuni infection were the most commonly etiological agents in GBS development in a meta-analysis [14]. However, this meta-analysis did not include the six cases of GBS subsequent to Hantavirus infection because they were single case reports.

In summary, we hold the opinion that our patient is a case of GBS (AMSAN) associated with Hantavirus infection. In intensive care settings, ICUAW should be suspected once the patients presented flaccid quadriparesis and neuromuscular respiratory failure, especially after recovery of sepsis, shock and multiorgan failure. Of note is that in patients with virus or Campylobacter jejuni infection, EMG should be performed to identify GBS. In that case, targeting therapy, like IVIG or PE, could be immediately initiated to get a better prognosis.
